# Incidental gastric diverticulum in a young female with chronic gastritis: A case report

**DOI:** 10.1016/j.ijscr.2019.11.030

**Published:** 2019-11-27

**Authors:** Eugene Richard Joweni Baloyi, David Morris Rose, Nolitha Makapi Tisetso Morare

**Affiliations:** University of Witwatersrand, Klerksdorp Tshepong Hospital Complex, Benji Oliphant Street, North West, South Africa

**Keywords:** CT, Computed Tomography, OGD, Oesophagogastroduodenoscopy, US, Ultrasound, NSAID, Non-Steroidal Anti-Inflammatory Drug, PPI, Proton-Pump Inhibitor, GD, Gastric Diverticulum, Gastrointestinal abnormalities, Diverticulosis, Dyspepsia, Congenital defects: oesophagogastroscopy

## Abstract

**Introduction:**

Gastrointestinal upset is a common presentation to surgical departments, often requiring investigation with endoscopy. Pathologies such as gastritis or ulcers are common culprits. Occasionally, rare or unusual pathologies, such as gastric diverticula as was seen in the case presented, are found.

**Case Presentation:**

A 26 year old female, with no known co-morbid conditions presented with a two week history of abdominal pain associated with nausea and vomiting. On further inquiry, she had one episode of blood stained vomiting, prompting investigation with an oesophagogastroduodenoscopy (OGD). Findings included diffuse haemorrhagic gastritis with a single outpouching measuring 1–2 cm in the gastric fundus. A gastric diverticulum was confirmed on barium swallow. Investigation with sonar and a Computed Tomography (CT) scan reported the stomach as normal.

**Conclusion:**

The patient was successfully treated non-operatively with proton pump inhibitor therapy for her concomitant gastritis. Gastric Diverticula are often associated with other gastric findings and their individual contribution varies from case to case.

**Discussion:**

Gastric Diverticula are the manifestation of a common condition in an unusual location. Their clinical implications vary from being insignificant to life threatening when complicated by haemorrhage, perforation or malignant transformation. The associated symptoms are non-specific and diagnosis may be challenging. The case highlights the importance of selecting appropriate imaging modalities for luminal structures, being only diagnosed in 2 (OGD, Swallow) of the four modalities (incl. ultrasound and CT scan) used. Treatment may be conservative or surgical and is patient dependent. Written consent and ethical approval was obtained. The work is reported in line with the SCARE criteria.

## Highlights

• Gastrointestinal diverticula, although common, may occur in unusual locations such as the gastric region.
• Gastric Diverticula usually present with vague abdominal symptoms and a high index of suspicion may be needed to make the diagnosis.
• Gastric Diverticula may be diagnosed using different modalities with varying results, the gold standard of which is OGD.
• Careful positioning (supine, left lateral decubitus, Trendelenburg) may augment contrast study findings.
• Gastric Diverticula are usually asymptomatic and benign but rare cases of serious complications such as perforation, bleeding and malignant transformation have been described.

## 1 Background

Gastric diverticula are rare worldwide with an incidence of 0.02 % in autopsy studies [[1], [2], [3], [4], [5]]. The diagnosis is based on a history of vague abdominal symptoms coupled with non-specific physical signs, which may mimic more prevalent gastrointestinal conditions [1,2]. For this reason, a high index of suspicion is required should one intend on making the diagnosis. Modalities used to facilitate this include: upper gastrointestinal contrast studies, OGD and Computed Tomography (CT) scan. Although these typically don’t require intervention, management for associated manifestations or complications (e.g. bleeding or perforation), may be medical or surgical [[1], [2], [3], [4]]. The following case is of a patient who presented with a gastric diverticulum diagnosed on OGD and contrast meal, at a secondary hospital in North West, South Africa.

## 2 Case presentation

A 26-year-old female presented, walking, to the emergency department with a 2 week history of abdominal pain radiating to the back. This was associated with a 2 day history of nausea and vomiting following an alcohol binge. On one episode the vomitus was noted to be blood stained. She had no prior medical, surgical or family history. She consumed alcohol regularly and had a binge on the two days prior. She consumed Non-Steroidal Anti-Inflammatory Drugs (NSAID) chronically and had an eight-pack-year history of smoking.

On arrival she had a blood pressure of 124/64 mmHg and was tachycardic with a heart rate of 118bpm. She had a temperature of 37 °C. Her urine dipstick and pregnancy tests were negative. On examination she had epigastric tenderness but her abdomen was soft and she had no signs of peritonitis. Her cardiovascular, respiratory and central nervous system examinations were unremarkable. She had no malaena or blood on rectal examination.

## 3 Investigations

She had normal electrolytes, septic markers, liver and renal function on formal bloods. Her amylase was 35 U/L. Her haemoglobin was 14.2 g/dl. Her abdominal and chest x-rays were normal.

An abdominal ultrasound (US) reported a normal pancreas, liver, spleen and kidneys. The gallbladder was normal with no thickening, pericholecystic fluid or calculi. There was no free intraperitoneal fluid noted or abnormal masses.

**Fig. 1:**
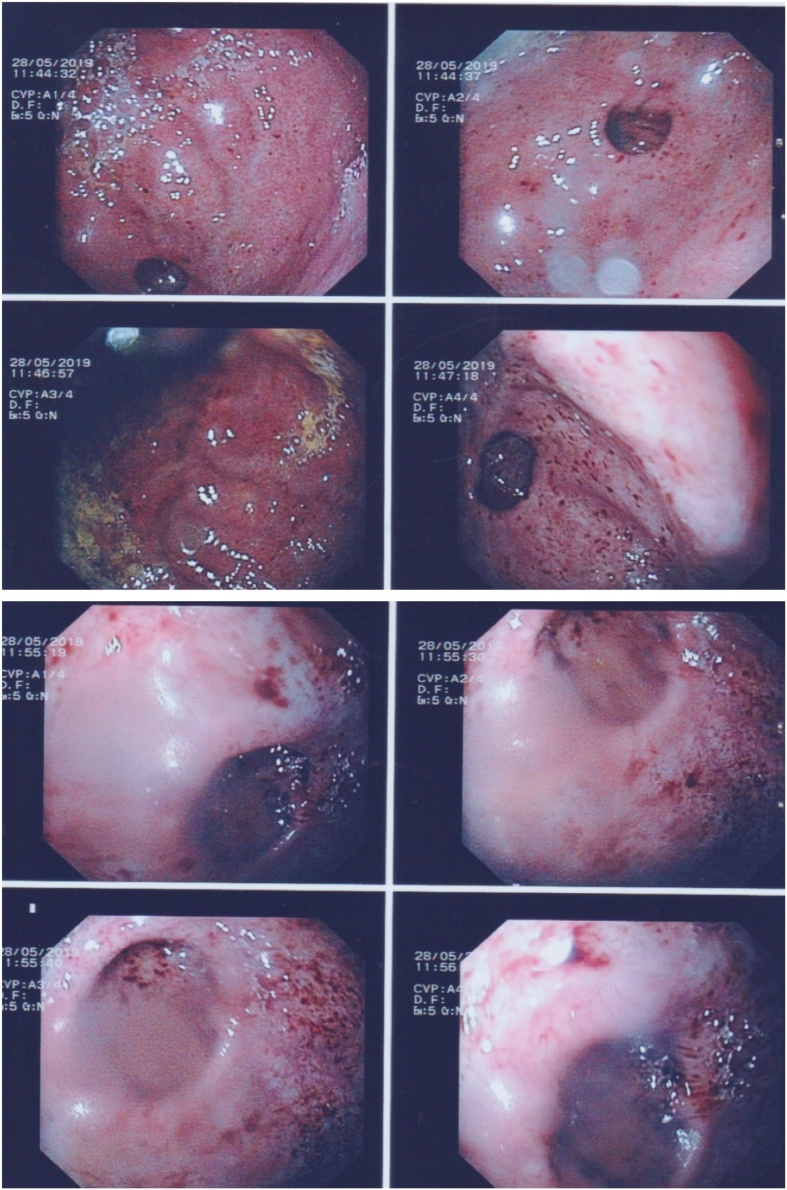
Images taken on Esophagogastroduodenoscopy demonstrating haemorrhagic gastritis with a single, regular, 1–2 cm outpouching of the gastric mucosa located in the gastric fundus.

She was subsequently sent for OGD, performed by the surgical registrar with supervision, which found diffuse haemorrhagic gastritis. A single outpouching estimated to measure 1–2 cm was noted in the gastric fundus, the mucosa was otherwise regular and there were no signs of perforation (Fig. 1). A biopsy was taken of the gastric mucosa to exclude ectopic tissue and helicobacter pylori as a cause of the gastritis.

**Fig. 2:**
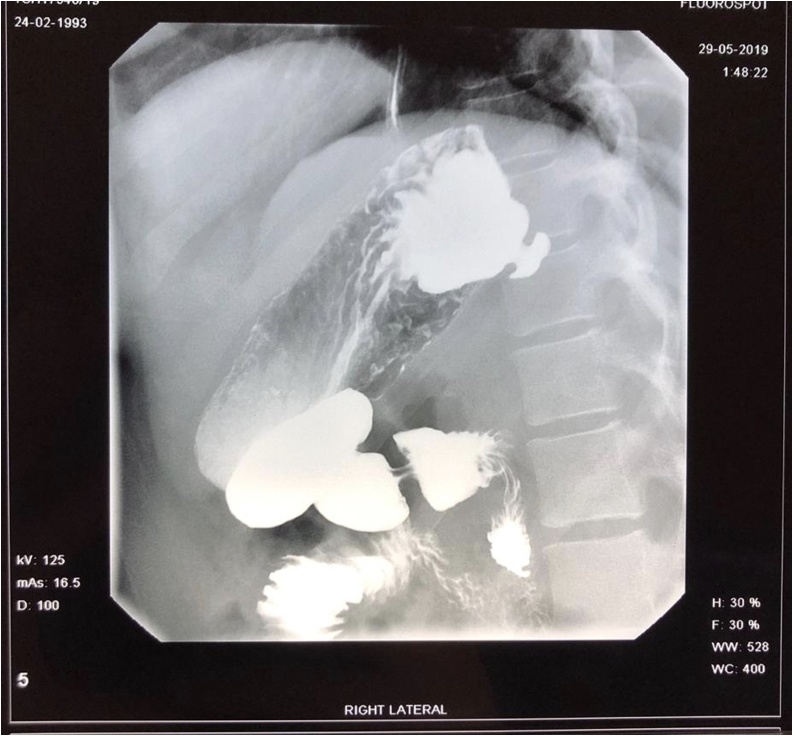
Right lateral view of barium contrast meal demonstrating a gastric diverticulum arising posteriorly in the gastric fundus, measuring 19 × 10 mm.

She was sent for a CT scan of her abdomen which was reported as normal. Based on her OGD findings, she was sent for a barium swallow. She was given 1 g of Calcium Carbonate effervescent tablets in order to assist with gastric distention. Informed by the literature, she was positioned supine with a slight left lateral tilt with the feet at a 30° incline (Trendelenburg) position [5]. The contrast swallow revealed a gastric diverticulum arising posteriorly from the lesser curvature of the fundus measuring 19 × 10 mm on the right lateral view (Fig. 2), there was no other abnormality in gastric mucosa or emptying.

Her histology from the biopsy taken at OGD revealed moderate chronic superficial gastritis with no helicobacter pylori organisms found and no intestinal metaplasia, dysplasia or malignancy.

## 4 Differential diagnosis

Several differential diagnoses were considered:

1. Peptic ulcer disease: this is based on her history of symptoms, physical examination and risk factors i.e. smoking, NSAID use, ethanol history. This was excluded on OGD.
2. Acute pancreatitis: this is based on the characteristic history of her pain together with a recent alcohol binge. This was excluded on formal blood results revealing normal amylase with no features of pancreatitis on sonar or CT scan.
3. Symptomatic cholelithiasis: this is based on her risk factors (female, increased BMI) as well as her presentation with upper abdominal pain. This too was excluded on sonar and CT scan.

## 5 Treatment

Prior to her gastroscopy, an Intravenous (IV) line was inserted and she was given fluid rehydration with crystalloid fluids (i.e. BALSOL) and given an immediate Intravenous dose of Pantoprazole 80 mg. Based on local treatment guidelines for gastritis, following the OGD she was started on a course of Proton Pump Inhibitor (PPI), omeprazole 20 mg orally 12 hourly for 4 weeks, and she was given Paracetamol 1 g orally 6hourly as analgesia. She had no further episodes of hematemesis while in hospital and was discharged 2 days later.

## 6 Outcome and follow-up

On follow up after a month, she remained asymptomatic. On clinical examination she had no pallor and had an unremarkable abdominal and per rectal examination. It was deduced that her abdominal symptoms were more likely attributable to her gastritis. She was counselled on the potential dangers of NSAID use and their contribution to her condition, smoking cessation and responsible drinking. She was advised to return for a repeat scope should symptoms recur, at which stage further intervention could be considered.

## 7 Discussion

A Gastric diverticulum (GD) is an outpouching of the gastric wall which most often occurs in the posterior wall of the fundus, as was demonstrated in the case above [5]. They are the least common gastrointestinal (GI) diverticula, with prevalence rates ranging from 0.04% (165/380,000) in upper GI contrast studies, 0.01–0.11% in OGD and 0.02% (6/29,900) in autopsy studies [4,5].

These may be classified as: congenital or acquired, true or false. The difference between true and false diverticula is based on the former containing all the layers of the gastric wall as opposed to false diverticula which do not. Congenital diverticula are typically true and constitute ¾ of all GD’s, while those that are acquired are typically false [5]. Congenital diverticula, are typically found along the dorsal wall of the fundus, while acquired diverticula are situated in or near the antrum [5]. The pathogenesis of false diverticula is based on forces which may be internal (an increase in the intraluminal pressure from Labour, Gastric Outlet Obstruction, prolonged vomiting etc.) known as “pulsion diverticula”, or external (contractile pulling forces from external adhesions, fibrosis etc.) known as “traction diverticula” [2,3]. Congenital typically result from developmental abnormalities during the foetal period [2,3].

GD may be asymptomatic but when they do occur, patients typically present with a long history of vague abdominal complaints such as: dyspepsia, upper abdominal pain, nausea, vomiting and early satiety. Due to the non-specific nature of the symptoms, these may be attributed to an array of other abdominal pathologies and a diagnosis is often made incidentally in the workup for such. In 1951, following the review of 49 symptomatic cases of GD, Palmer suggested that symptomatic diverticula are usually seen in the presence of another gastric abnormality such as ulceration or gastritis (as was seen in our case) [5,6]. This brings into question, the clinical significance of gastric diverticula in isolation. It also highlights the need to look for another pathology either clinically (where changes are macroscopically apparent) or with biopsy. While most presentations are benign, patients may present with more serious symptoms following complications such as bleeding and perforation [[1], [2], [3], [4], [5]]. Cases of unusual associations i.e. obstruction and malignancy have been reported in the literature [7,8]. Jayarajah et al. reported a case of a patient presenting with Gastric Outlet Obstruction (GOO) who was noted to have a pre-pyloric diverticulum on OGD [7]. Fork et al. reported the case of a 77 year old man known with a GD, who was later found to have a malignant polypoid lesion developing within it 11 years later [8]. For this reason, any unusual features in a GD should prompt further investigation.

The gold standard investigation modality is OGD. This not only confirms the diagnosis but co-existing pathologies may be identified and unusual features (irregular mucosa, ulceration etc.) may be biopsied. Another modality is an contrast meal, but this carries a 5% false negative rate [6]. This may be improved with meticulous positioning as was conducted in our case as informed by the literature (prone, Trendelenburg, left decubitus) [5]. CT scan is another option, but this is less sensitive and may lead to misdiagnosis such as adrenal pathology [5]. Our case made use of all three recommended modalities as well as ultrasonography for comparison. Of these CT and ultrasound failed to demonstrate the diverticulum, which was clearly visualised on OGD and contrast swallow. This highlights the strengths and weaknesses of these modalities when evaluating subtle, intraluminal gastric pathologies.

Management of GD is patient dependant based on the severity of the presenting complaints, size, presence of complications and patient profile. Medical treatment with the use of protein pump inhibitors, antacids or antihistamines, have been reported to provide a degree of symptomatic relief without resolving the underlying pathology [4,5]. Surgical management is reserved for large (>4 cm), symptomatic or complicated GD with both laparoscopic and open resection being effective [2,5]. Surgery ranges from diverticulum invagination to partial gastrectomy [3]. The patient in the case presented had concomitant gastritis, experienced symptomatic relief following treatment with a PPI and the diverticulum was small (<4 cm). As such, surgical management was deemed unnecessary at this stage. Regardless, the unusual OGD finding presented an interesting opportunity for learning and reflection.

In conclusion, gastric diverticula represent a common condition in an unusual location. The symptoms of it vary and the clinical significance more so. Our patient was found to have concomitant gastritis which is in keeping with the findings by Palmer et al. that these diverticula are often associated with other pathologies [6]. She was investigated with 4 different modalities (OGD, Ultrasound, CT scan and contrast meal) and the diverticulum was only noted in 2 of these consistent with OGD being the gold standard in the literature. Following treatment of her gastritis her symptoms improved and therefor they were attributed to this. Despite our conclusion that the diverticulum in itself was likely of no clinical significance for this patient, the case offers an opportunity for reflection of anatomy and rare variations thereof.

## Patient’s perspective

“Coming to the hospital I was very worried, especially when I started vomiting blood. I was worried that this pain and vomiting was all related to the alcohol I had taken. I had heard from people that taking certain painkillers could give you ulcers but I had also being told that it rarely happens. I agreed to have all the investigations done because I wanted to know what was wrong with me and to get better. I find it interesting that I have such a rare condition but happy that it is not life threatening”

## Declaration of Competing Interest

There are no conflicts of interest to declare for any of the authors.

## Funding

No funding was received to conduct this research

## Ethical approval

Ethics was granted from the Human Research Ethics Committee at the University of Witwatersrand. The approval was unconditional with the following reference number: M190583.

## Consent

Written informed consent was obtained from the patient for publication of this case report and accompanying images. Confidentiality was maintained in the writing of this report.

## Author contribution

Nolitha Morare- Conceptualization, Validation, Investigation, Writing – Original Draft, Writing – Review & Editing, Supervision

Eugene Baloyi- Writing, Original Draft, Resources

David Rose- Software, Writing – Review & Editing, Visualization

## Registration of research studies

This is a case report

## Guarantor

Nolitha Morare (corresponding author) is the primary guarantor. All of the authors however, accept full responsibility for the work.

## Provenance and peer review

Not commissioned, externally peer-reviewed
